# Fatal Granulomatosis with Polyangiitis Presenting as Medial Longitudinal Fasciculus Syndrome with Gastrointestinal Perforation: An Autopsy Case Report

**DOI:** 10.31662/jmaj.2025-0255

**Published:** 2025-11-21

**Authors:** Saori Inoue, Takashi Kamiya, Reiichiro Fujita, Kaoru Yoshida, Aya Morita, Hiroko Yasuda

**Affiliations:** 1 Department of Internal Medicine, Meitetsu Hospital, Nagoya, Japan

**Keywords:** antineutrophil cytoplasmic antibody-associated vasculitis, granulomatosis with polyangiitis, medial longitudinal fasciculus syndrome, gastrointestinal perforation

## Abstract

We report a rare and fatal case of granulomatosis with polyangiitis (GPA) initially presenting as medial longitudinal fasciculus (MLF) syndrome. A 64-year-old man with a history of duodenal ulcer and hypertension presented with diplopia. Neurological examination revealed impaired adduction of the left eye with preserved convergence, consistent with MLF syndrome. Brain magnetic resonance imaging showed multiple high-intensity lesions, including one in the left dorsal pons. Laboratory testing revealed an elevated PR3 antineutrophil cytoplasmic antibody level of 205 IU/mL. A kidney biopsy on day 18 revealed crescentic necrotizing glomerulonephritis, confirming the diagnosis of GPA. Corticosteroid pulse therapy was initiated, but gastrointestinal perforation occurred on day 26, requiring extensive gastrectomy. Pathology revealed ulcerative changes and neutrophil infiltration, but no vasculitic lesions. Because of hepatic dysfunction from hepatitis C virus infection, escalation of immunosuppressive therapy was not feasible. On day 111, the patient developed a second gastrointestinal perforation, leading to sepsis and death on day 136. Autopsy revealed duodenal perforation and multiple cerebral infarctions corresponding to prior magnetic resonance imaging findings. No definitive vasculitic lesions were identified. This case highlights an unusual central nervous system onset of GPA as MLF syndrome. Antineutrophil cytoplasmic antibody-associated vasculitis should be considered even in patients with atypical neurological manifestations, and prompt diagnosis and treatment are essential.

## Introduction

Antineutrophil cytoplasmic antibody (ANCA)-associated vasculitis (AAV) is a systemic disease that primarily affects small- to medium-sized vessels. Patients presenting with necrotizing granulomatous inflammation, proteinase 3-ANCA (PR3-ANCA) positivity, and involvement of the lungs, ear, nose, throat, and kidneys are classified as having granulomatosis with polyangiitis (GPA) ^(1), (2)^. In GPA, central nervous system (CNS) and gastrointestinal (GI) involvement are relatively rare ^(3)^. Medial longitudinal fasciculus (MLF) syndrome is an ocular movement disorder caused by microvascular brainstem lesions. Here, we report a rare case of GPA in a patient who initially presented with MLF syndrome.

## Case Report

**Figure 1.**
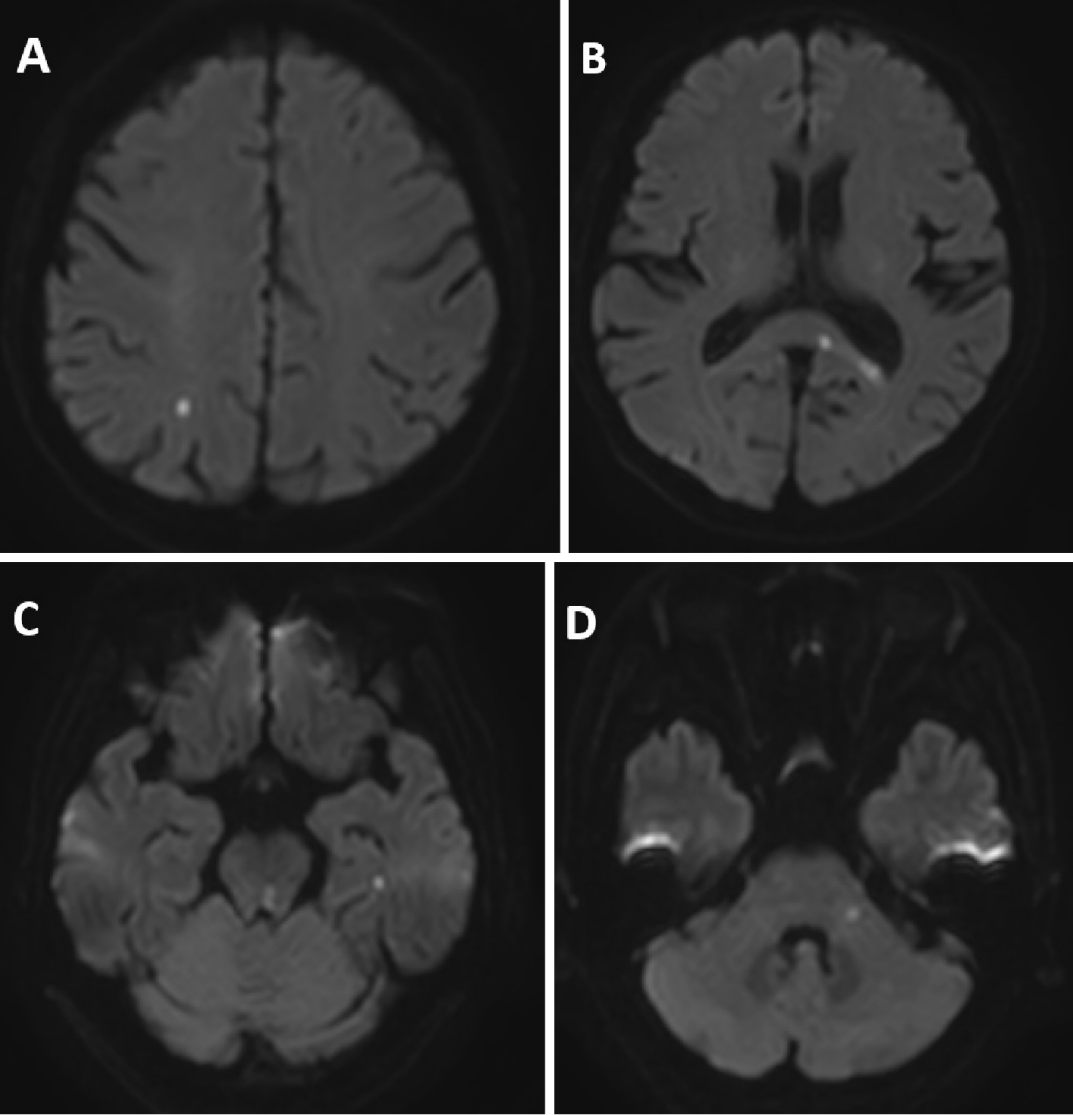
Diffusion-weighted magnetic resonance images obtained on admission showing multiple hyperintense lesions suggestive of acute infarctions. (A) Right parietal lobe. (B) Corpus callosum. (C) Left temporal lobe and dorsal part of the left pons, including the medial longitudinal fasciculus. (D) Left middle cerebellar peduncle.

The patient was a 64-year-old man with a medical history of duodenal ulcer and hypertension, managed with amlodipine 5 mg and olmesartan 10 mg. On day 1 of illness, he presented to the emergency department with diplopia. Neurological examination revealed impaired adduction of the left eye with preserved convergence, leading to a diagnosis of MLF syndrome. He was alert and oriented, with no paralysis, no edema, and clear breath sounds. Brain magnetic resonance imaging (MRI) showed multiple hyperintense lesions in the MLF and other regions (Figure 1). Blood tests revealed elevated PR3-ANCA (Table 1). Chest and abdominal computed tomography showed no abnormalities.

**Table 1.** Initial Laboratory Findings on Admission.

Laboratory Findings			
Parameter	Result	Reference Range	Unit
Albumin	2.6	4.1-5.1	g/dL
Blood Urea Nitrogen	36	8-20	mg/dL
Creatinine	1.96	0.65-1.07	mg/dL
CRP	7.09	0.00-0.14	mg/dL
AST	27	13-30	U/L
ALT	25	10-42	U/L
ALP	167	106-322	U/L
LDH	204	124-222	U/L
Sodium	129	138-145	mEq/L
Potassium	3.8	3.6-4.8	mEq/L
Chloride	95	101-108	mEq/L
Blood Glucose	129	73-109	mg/dL
WBC	9,540	3,300-8,600	/μL
RBC	4.19 × 10^6^	4.35-5.55×10^6^	/μL
Hemoglobin	11.7	13.7-16.8	g/dL
Platelets	283 × 10^3^	158-348×10^3^	/μL
**Serology and Immunology**			
**Test**	**Result**	**Reference Range**	**Unit**
Antinuclear Antibody	<40	<40	titer
PR3-ANCA	205.0	―	U/mL
MPO-ANCA	40.7	―	U/mL
Anti-cardiolipin Antibody	<8	<8	U/mL
FDP	6.3	≤5	μg/mL
D-dimer	2.0	≤1	μg/mL
HCV Antibody	Positive	Negative	―
Blood Culture	Negative	Negative	―
**Urinalysis**			
**Parameter**	**Result**	**Reference Range**	**Unit**
Urine Protein	+1	Negative	―
Urine Protein/Creatinine Ratio	0.92	<0.15	g/g·Cr
Urine Glucose	Negative	Negative	―
Occult Blood in Urine	+3	Negative	―
β_2_-Microglobulin	15,300	<230	μg/L
NAG	17.5	0.7-11.2	U/L

Abbreviations: ALP: alkaline phosphatase; ALT: alanine aminotransferase; AST: aspartate aminotransferase; CRP: C-reactive protein; D-dimer: fibrin degradation product; FDP: fibrin/fibrinogen degradation products; HCV: hepatitis C virus; LDH: lactate dehydrogenase; MPO-ANCA: myeloperoxidase–anti-neutrophil cytoplasmic antibody; NAG: N-acetyl-β-D-glucosaminidase; PR3-ANCA: PR3–anti-neutrophil cytoplasmic antibody; RBC: red blood cell count; WBC: white blood cell count; β2-MG: beta-2 microglobulin.

Following admission, treatment with argatroban was initiated, and the eye movement disorder improved by day 7. On day 17, renal function deteriorated. A renal biopsy was performed on day 18, and the patient was diagnosed with GPA based on findings of crescentic necrotizing glomerulonephritis (Table 2). Although immunofluorescence revealed immunoglobulin M deposition, crescentic necrotizing glomerulonephritis with fibrin deposition and arteritis was observed, consistent with ANCA-associated vasculitis. Immune complex deposition has been reported in a subset of ANCA-associated glomerulonephritis cases and does not exclude the diagnosis of GPA ^(4)^.

**Table 2.** Renal Biopsy.

Marker	Result
IgG	Granular deposition in the mesangium
IgA	Granular deposition in the mesangium
IgM	Homogeneous deposition in the capillary walls
C1q	Granular deposition in the mesangium
C3c	―
C4	―

Renal biopsy confirmed crescentic necrotizing glomerulonephritis, supporting the diagnosis of granulomatosis with polyangitis. Infiltration of neutrophils and fibrin deposition were observed in small arteries, indicative of arteritis. Immunofluorescence staining showed significant IgM deposition. Abbreviations: C1q: complement component 1q; C3c: complement component 3c; C4: complement component 4; IgA: immunoglobulin A; IgG: immunoglobulin G; IgM: immunoglobulin M.

According to guidelines, antiviral therapy is prioritized for patients with chronic hepatitis C ^(5)^. However, because of the disease severity and urgency in this case, corticosteroids were administered. Corticosteroid pulse therapy was initiated on day 23 (Table 3). Loxoprofen 60 mg was given once daily, but a proton pump inhibitor was not co-administered. On day 26, the patient developed upper GI perforation and underwent subtotal gastrectomy. The resected specimen showed ulcerative changes, but no pathological findings suggestive of AAV.

**Table 3.**
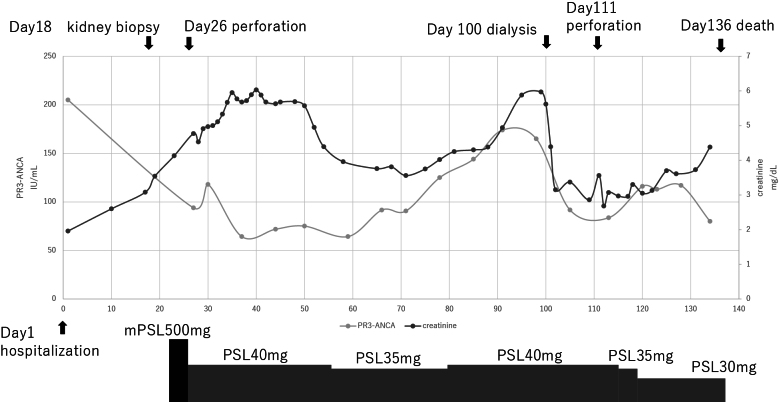
Clinical Course of PR3-ANCA and Serum Creatinine Levels from Day 1 (Day of Admission). Thistable summarizes the patient’s clinical course, showing serial changes in PR3-ANCA titers and serum creatinine levels. It also includes the timing and dosage of corticosteroid therapy, including intravenous mPSL and oral PSL, administered during hospitalization. Abbreviations: mPSL: methylprednisolone pulse therapy; PR3-ANCA: PR3–anti-neutrophil cytoplasmic antibody; PSL: prednisolone.

The prednisolone dosage was adjusted based on the ANCA levels. Hepatitis C virus RNA was positive, and viral reactivation was observed (Table 4). Because of severe acute liver injury, cyclophosphamide was deemed unfeasible. On day 111, retroperitoneal emphysema was noted, and another GI perforation occurred. Despite treatment, the patient developed sepsis and died on day 136.

**Table 4.** Laboratory Findings on Day 61 Showing Reactivation of Hepatitis C Virus and Liver Dysfunction.

Test	Result	Reference Range	Unit
HCV-RNA	5.6	Undetectable	log IU/mL
Bilirubin	0.51	0.4-1.5	mg/dL
AST	176	13-30	U/L
ALT	523	7-23	U/L
ALP	530	100-340	U/L
γ-GTP	210	10-47	U/L

Abbreviations: ALP: alkaline phosphatase; ALT: alanine aminotransferase; AST: aspartate aminotransferase; HCV-RNA: hepatitis C virus ribonucleic acid; γ-GTP, gamma-glutamyl transpeptidase.

Cytomegalovirus antigenemia was positive, but antiviral treatment was not initiated because of the lack of supportive findings.

Autopsy revealed duodenal anastomotic perforation, and the cause of death was determined to be sepsis-induced circulatory failure. Multiple cerebral infarctions were also observed. No definitive pathological evidence of vasculitis was found.

## Discussion

CNS involvement in GPA is uncommon but is characterized by multiple bilateral microvascular lesions ^(6), (7)^. MLF syndrome typically results from small-vessel lesions in the midbrain and may be attributable to vasculitis. In this case, brain MRI showed multiple hyperintense lesions, suggesting ischemic damage due to the microvasculitis characteristic of GPA.

CNS involvement in AAV tends to be associated with a higher Birmingham Vasculitis Activity Score and is considered a poor prognostic factor ^(8)^. The Birmingham Vasculitis Activity Score in this case was high at 39. Although the patient initially responded to treatment, deterioration of his general condition and limitations in immunosuppressive therapy may have affected the prognosis.

In AAV, CNS involvement may occur through the following three mechanisms ^(7)^: inflammation and occlusion of small- to medium-sized cerebral vessels due to systemic vasculitis, invasion or compression by granulomatous lesions from adjacent structures, and formation of de novo granulomatous lesions within the CNS. In this case, no adjacent granulomatous lesions were observed. Contrast-enhanced MRI showed no evidence of tumor-like lesions in the CNS. Therefore, we consider the infarctions to be secondary to vasculitis-associated vascular injury.

GI perforation in GPA is rare and most commonly involves the ileum ^(9)^. While vasculitic damage is the primary cause of GI perforation in AAV, no pathological findings suggestive of vasculitis were observed in either the surgical or autopsy specimens. The combination of a history of peptic ulcer, use of loxoprofen, and high-dose corticosteroid therapy likely contributed to the perforation. According to clinical guidelines, prophylactic administration of proton pump inhibitors should be considered in patients receiving corticosteroids and/or NSAIDs to prevent gastrointestinal mucosal injury ^(10)^. At the time of the second GI perforation, ANCA levels had decreased, suggesting corticosteroid-induced tissue fragility rather than active vasculitis.

The absence of typical vasculitic lesions may reflect a positive treatment response ^(11)^. In this case, impaired liver function limited the use of cyclophosphamide, making rituximab a potential alternative. However, rituximab was not widely used in Japan in 2019, particularly in patients with hepatic dysfunction, although its use might have improved the prognosis.

Despite a thorough literature search, no reports of vasculitis presenting as MLF syndrome were found. Even in patients with atypical CNS symptoms, clinicians should consider the possibility of AAV and pursue early diagnosis and appropriate therapeutic interventions, including rituximab initiation.

## Article Information

### Acknowledgments

The authors thank Angela Morben, DVM, ELS, from Edanz (https://jp.edanz.com/ac), for editing a draft of this manuscript.

### Author Contributions

Patient care and contribution to writing, design, and editing of the manuscript: Saori Inoue. Patient care and assistance with manuscript editing: Takashi Kamiya and Hiroko Yasuda. Supervision and guidance during manuscript preparation: Reiichiro Fujita, Kaoru Yoshida, and Aya Morita.

### Conflicts of Interest

None

### Informed Consent

Written informed consent was obtained from the patient’s family for the anonymous publication of this case report.
